# How to strengthen societal impact of research and innovation? Lessons learned from an explanatory research-on-research study on participatory knowledge infrastructures funded by the Netherlands Organization for Health Research and Development

**DOI:** 10.1186/s12961-024-01175-x

**Published:** 2024-07-08

**Authors:** Wija Oortwijn, Wendy Reijmerink, Jet Bussemaker

**Affiliations:** 1 Leiden University Medical Centre//Health Campus Den Haag, The Hague, The Netherlands; 2 Radboud University Medical Center, Health Evidence, Nijmegen, The Netherlands; 3 ZonMw, The Hague, The Netherlands; 4 Institute of Public Administration, Leiden University, Leiden, The Netherlands

**Keywords:** Impact, Participatory knowledge infrastructures, Collaboration, Health research funder, Co-creation, Productive interactions

## Abstract

**Background:**

Scientific research and innovation can generate societal impact via different pathways. Productive interactions, such as collaboration between researchers and relevant stakeholders, play an important role and have increasingly gained interest of health funders around the globe. What works, how and why in research partnerships to generate societal impact in terms of knowledge utilisation is still not well-known. To explore these issues, the Netherlands Organization for Health Research and Development (ZonMw) initiated an exploratory research-on-research study with a focus on participatory knowledge infrastructures (PKIs) that they fund in the field of public health and healthcare. PKIs are sustainable infrastructures in which knowledge production, dissemination and utilisation takes place via committed collaboration between researchers and stakeholders from policy, practice and/or education. Examples are learning networks, academic collaborative centres, care networks and living labs. The aim of the study was twofold: to gain insights in what constitutes effective collaboration in PKIs; and to learn and improve the research governance, particularly of ZonMw as part of their dissemination and implementation activities.

**Methods:**

During 2020–2022, we conducted a literature review on long-term research partnerships, analysed available documentation of twenty ZonMw-funded PKIs, surveyed participants of the 2021 European Implementation Event, interviewed steering committee members, organized a Group Decision Room with lecturers, and validated the findings with key experts.

**Results:**

We identified eight mechanisms (‘how and why’) that are conditional for effective collaboration in PKIs: transdisciplinary collaboration; defining a shared ambition; doing justice to everyone’s interests; investing in personal relationships; a professional organisation or structure; a meaningful collaborative process; mutual trust, sufficient time for and continuity of collaboration. Several factors (‘what’) may hinder (e.g., lack of ownership or structural funding) or facilitate (e.g., stakeholder commitment, embeddedness in an organisation or policy) effective collaboration in research partnerships.

**Conclusion:**

To use the study results in policy, practice, education, and/or (further) research, cultural and behavioural change of all stakeholders is needed. To facilitate this, we provide recommendations for funding organisations, particularly ZonMw and its partners within the relevant knowledge ecosystem. It is meant as a roadmap towards the realisation and demonstration of societal impact of (health) research and innovation in the upcoming years.

**Supplementary Information:**

The online version contains supplementary material available at 10.1186/s12961-024-01175-x.

## Background

Scientific research, from fundamental research to implementation projects, is essential for tackling societal issues such as climate change and global health. Whether it concerns sustainability, social inequality or an aging population, various types of knowledge and perspectives are always needed to properly understand and address the problem [1]. This raises questions about what this requires from researchers involved; in the articulation of the problem definition and possible co-creation with non-scientific partners; in the way they perform the research, collaborate and communicate with other partners, and in the way they maximize the societal impact of their research. Since the early 2000s, achieving societal impact became high on the research agenda [2], and it became a task of universities next to research and education [3].

Several frameworks have been developed (e.g., payback model, (hybrid) Research Impact Framework, health economics models, contribution mapping) [4–8] and several studies have been conducted to assess the societal impact of research and innovation, e.g., [9–11]. From these studies it is known that successful knowledge utilisation involves so-called productive interactions [12]. These interactions include (1) direct interactions such as collaboration between researchers and relevant stakeholders (e.g., funders, practitioners, policy makers, and citizens); (2) indirect interactions such as generation of useful knowledge products and targeted dissemination and implementation activities; and (3) financial interactions such as co-funding. Scientific research can generate impact via different routes and through different mechanisms, depending on the (desired) type of knowledge utilisation and productive interactions [13]. Productive interactions are particularly at play in so-called participatory knowledge infrastructures (PKIs). PKIs are sustainable partnerships in which knowledge production, dissemination and utilisation take place via committed collaboration between researchers and different stakeholders (i.e., those active in policy, practice and/or education). Examples of PKIs are learning networks (LN), academic collaborative centres (ACN), disease-related/care networks (CN), consortia (C), knowledge portals (KP) and living labs (LL).

Productive interactions have increasingly gained interest of (health) funders around the globe [4, 6]. One of these funders, the Netherlands Organization for Health Research and Development (ZonMw), funds and promotes (the use of) health research and care innovation throughout the entire knowledge chain from fundamental research to implementation projects. ZonMw defines impact in terms of knowledge utilisation—the use of results of projects and programmes for value-creation in policy, practice, education and/or (further) research [14]. ZonMw’s task is achieving and demonstrating impact of funded research for reasons of accountability, analysis, and optimizing resource allocation. Part of this task is learning about routes to knowledge utilisation, the effectiveness of their policies towards, and added value of strengthening impact. Routes to knowledge utilisation are so-called impact pathways, and can be identified by asking “what works, how and why?”.

For 20 years, ZonMw has been funding PKIs as part of building capacity: investing in people and structures that enable the release of findings, dissemination, knowledge exchange/partnering, and/or implementation of research evidence [15], which is internationally quite unique. However, what works, how and why in terms of collaboration with regard to these infrastructures is still not well-known [16, 17].To explore these issues, ZonMw initiated a research-on-research study.

## Methods

The aims of this explanatory study were to gain insights in what constitutes effective collaboration in PKIs to accelerate knowledge utilisation; and to learn and improve the research governance of funders, including ZonMw as part of their dissemination and implementation activities [15]. The extent to which collaboration actually leads to effective use of knowledge in practice, policy, education and/or further research was not studied. Also, a detailed analysis by type of PKI (e.g., the number and type of participating organisations, the selection procedure, the personal characteristics of participants and type of governance) was beyond the scope of this study.

The study was executed by ZonMw staff in close collaboration with researchers from Leiden university medical centre/University of Leiden and overseen by a steering committee of six experts (all professors) from different disciplines relevant to the study.

From December 2020 until December 2022, we undertook several research activities to address both aims described above. We conducted a literature search focused on effective mechanisms for collaboration in research partnerships, that would also apply to PKIs. The publications of Kaats and Opheij [18] on conditions for collaboration in partnerships and Muhonen et al. [13] on impact pathways using productive interactions were used as a starting point as they were identified in earlier work that we conducted on measuring the impact of research,^1^ and we used snowballing to retrieve additional relevant articles. This initial search was supplemented by a systematic search strategy on facilitators and barriers of long-term collaboration using PubMed, Google (Scholar) and grey literature. The following search terms related to knowledge collaborations as identified in the initial publications were used: interdisciplinary, transdisciplinary, productive interactions, impact pathway, patient and public involvement, citizen science, health research, knowledge transfer and research partnerships. We included articles published between 1 January 2005 and 1 August 2022 that were available in English and/or Dutch. The literature review served as an input for analysing selected PKIs regarding what may hinder or facilitate effective collaboration and for categorizing recommendations on how to improve the research governance of funders. The categorization of recommendations is based on Brian Nosek’s model for cultural and behavioural change [19]. The model consists of five hierarchical levels of interventions for change: (1) it starts with creating an infrastructure, for example for data sharing; (2) to facilitate the change, data sharing needs to be made easy to use and integrated in existing practices; this is further enhanced by (3) addressing norms (e.g. regular knowledge sharing will make desired behaviour visible); (4) rewarding incentives (e.g. long-term financing of PKIs will nudge those involved to focus on long term outcomes and impact); and (5) policies (e.g. Open Science requirements). This model was chosen as it aligns well with our (systems) perspective in which there are interactions between micro (individual), meso (organization) and macro level (environment).

To complement and validate the results of the literature review a short online survey was developed and distributed via the ZonMw website to participants of the European Implementation Event in May 2021, which includes a broad range of professionals in the field of implementation of health research within Europe. The questions focused on identification of underlying mechanisms of effective collaboration in PKIs; recommendations for generating effective collaboration and also included questions on the role that funders should play in accelerating effective collaboration between researchers and stakeholders.

Based on an initial inventory by and in consultation with ZonMw implementation experts, 20 ZonMw-funded PKIs were selected for further analysis (see Table 1).

**Table 1:** Overview of PKIs included in the study

	Type	Domain	Name PKI (in Dutch)	Brief description
1	ACN	Disability care	Intellectual Disabilities (Verstandelijke Beperkingen)	6 ACN united in an association (more networks are connected to this)
2	ACN	Prevention	Consortium Integrated approach to obesity (Integrale aanpak Overgewicht)	5 ACN
3	ACN	Prevention	Public Health (Publieke Gezondheid)	11 ACN, currently independent organization
4	ACN	Elderly care	Elderly care (Ouderenzorg)	6 ACNs and an umbrella partnership
5	ACN	Youth	Youth (Jeugd)	ACN Youth (2009–2017–6 ACN) ACN Transformation Youth (2015–2020–13 ACN) ACN Regional knowledge networks Youth (2020–2024—15 ACN)
6	LN	Youth	Youth (Jeugd)	Link with ACN Youth—5 LN
7	LN	Mental health care	Action program Local initiatives for disordered persons (actieprogramma Lokale initiatieven voor mensen met verward gedrag)	5 educational institutions and 1 mental health institution
8	LN	Elderly care	Visible Link II (Zichtbare Schakel II)	11 LN, of which 1 has evolved into 1 ACN/LN District Nursing
9	LN	Elderly care/long-term care	District nursing (Wijkverpleging)	1 ACN (“Zorg voor beter”) has been financed via the ZonMw programme Zichtbare Schakel II
10	CN	Elderly care	Coherent care for the elderly / network care (Samenhangende Ouderenzorg/ Juiste Zorg op Juiste plek)	Support for local networks that facilitate, further develop and sustain integrated care for the elderly. In addition to the ZonMw-funded local networks there exists more than 1000 integrated networks for elderly care in place
11	CN	Long-term care/mental health care	Knowledge networks for specific target groups in long-term care	5 specific target groups; Knowledge network Korsakov; national NAH+ knowledge network; Huntington knowledge network Netherlands; Expertise center SGLVG De Borg (behaviour or psychological problems); healthcare institution Nieuw Unicum (MS patients)
12	CN	Curative care	Regional Oncology Networks	7 regions; Multidisciplinary in nature, thematic consultation of all care providers, patient associations, (national) organizations involved in oncology
13	CN	Curative care Elderly care/long-term care	Consortia Palliative Care Netherlands	7 consortia plus one national umbrella organization. Involvement of home care organizations, nursing homes; In addition to the ZonMw-funded networks there are at least 65 palliative care networks in place
14	CN	Disability care	Just Special (Gewoon Bijzonder)	12 (knowledge) networks aimed at health, behaviour, and participation
15	CN	Prevention	Make room for health (Maak ruimte voor gezondheid)	7 regional consortia
16	C	Prevention	Microplastics & Health (Microplastics & Gezondheid)	1 consortium MOMENTUM
17	C	Curative care Youth	Networks Pregnancy and Birth/Network Regional Consortia Maternity Care (Netwerken Zwangerschap en Geboorte/Netwerk Regionale Consortia Geboortezorg)	9 consortia, Collaboration with youth health care
18	KP	Disability care	Disability sector (Gehandicaptensector)	Meeting place to exchange knowledge and experience—physically during meetings, in network meetings or at the annual Knowledge Market, and digitally via website or social media
19	KP	Elderly care/long-term care Curative care (primary care)	Care for better (Zorg voor beter)	Digital meeting place (knowledge bundling), link with LN District nursing
20	LL	Prevention	Sports and Movement (Sport en Bewegen)	Co-creation: inventing and testing innovations in the user's actual environment; concerns 16 (8 large and 8 medium-sized) municipalities

*ACN* academic collaborative centres, *LN* learning networks, *CN* disease-related/care networks, *C* consortium, *KP* knowledge portal, *LL* living labs

To identify and describe the potential barriers and facilitators in the selected PKIs, we developed an analysis tool (template) based on the literature review (see Additional file 1). The tool was independently piloted by two authors (WO and WR) on one randomly chosen PKI. The pilot led to some minor revisions of the analysis tool, which includes the following elements:

- Description of the type of PKI (content/purpose, relationships/involved, structure/process);
- Barriers to collaboration (e.g. lack of ownership by knowledge users; insufficient funding or knowledge transfer; researchers' ‘hobbyhorses’; poor timing of results; power relations and conflicts of interest);
- Facilitators to effective collaboration (e.g. the role of project leaders; shared interests and goals; combination of ‘types’ of knowledge; stakeholder commitment; embedding in the organisation; translation of knowledge);
- Role of ZonMw as funder.

We then used the tool to analyse a large variety of available documentation from a variety of perspectives that were available for each PKI such as annual reports from PKI coordinating teams, internal and external evaluations, notes from ZonMw on the progress, and specific websites coordinated by PKIs, as well as other stakeholders. In addition, we reviewed whether specific underlying mechanisms could be identified in the different types of PKIs studied.

Subsequently, we conducted a digital Group Decision Room (GDR) with five lecturers (professors of practice-based research, affiliated to different universities of applied science) in May 2022. The lecturers have been selected from the platform of lecturers applied science in the field of healthcare and care by relevant contact persons of ZonMw. The GDR was moderated by an external expert, and we discussed the added value of practice-based research regarding collaboration, especially in PKIs; what challenges the lecturers encounter in participating in PKIs, and what measures would help to prevent/address this.

The interim results were discussed periodically with ZonMw implementation experts, as well as during meetings of the Ensuring Value in Research (EViR) Funders' Forum, the Netherlands Implementation Collaborative (NIC: a professional network for implementation scientists and implementation specialists in the Netherlands), and the Impact Alliance, a network of Dutch professionals with an interest in the societal impact of research. The interim results were also presented and discussed at a number of international conferences, including the European Implementation Event (EIE, May 2021), the Fifth Fuse International Conference on Knowledge Exchange in Public Health (June 2022) and the Advancing and Evaluating the Societal Impact of Science (AESIS) Conference (June 2022). These discussions primarily served to validate findings. The results have been published in a study report (in Dutch) for ZonMw, and this article is based on that report [20].

## Results

Despite the difficult comparability of the PKIs because they have their own set-up, approach and goals, as well as the risk of ‘narrative bias’ due to the variety of source information, the study adds valuable insights of what mechanisms (‘how and why’) are conditional for effective collaboration in order to obtain impact.

Based on the literature review, we identified eight mechanisms (‘how and why’) that are conditional for effective collaboration within PKIs. Without these mechanisms, it is plausible that collaboration will not be established or will not lead to any or less sustainable impact.

As described in the methods section, the starting point for identifying mechanisms was the publication of Kaats and Opheij [18] about collaboration between individuals and organisations in the public domain. Based on interdisciplinary scientific research as well as applied research, Kaats and Opheij distinguish five conditions for effective collaboration:

- Defining a shared ambition
- Doing justice to everyone's interests
- Investing in personal relationships (formal / non-formal)
- Having a professional organisation or structured process in place
- Having a meaningful process oriented towards collaboration (right steps, right sequence, win/win process, dialogue)

In addition to these mechanisms, we found in the literature that there is also a need for *mutual trust* between partner(s), as well as *sufficient time for and continuity of the collaboration* [21], adding up to seven mechanisms. The online survey of participants of the European Implementation Event 2021 yielded similar findings, although the number of respondents was low (*n* = 4). Another key publication, i.e., Muhonen et al. [13], studying mechanisms through which collaboration in the field of social sciences and humanities leads to societal impact, also came to similar conclusions. Finally, *transdisciplinary collaboration*—also referred to as knowledge co-creation—was found in the additional literature review to generate impact in (complex) knowledge and innovation processes, such as PKIs [13, 22]. This totals to eight different mechanisms that—in conjunction with each other—are decisive for effective collaboration (see Table 2).

**Table 2:** Mechanisms for effective collaboration in PKIs

1	Transdisciplinary collaboration
2	Defining a shared ambition
3	Doing justice to everyone’s interests
4	Investing in personal relationships
5	Mutual trust
6	Professional organisation or structure
7	Meaningful process of collaboration
8	Sufficient time for and continuity of collaboration

Based on the literature review, it becomes clear that all the mechanisms can either be enhanced or hindered by a combination of factors that relate to individuals involved in the partnership, their interrelationships and/or the working environment. For example, a shared set of values and ground rules, shared ownership for goal achievement, and activities supporting knowledge functions (measuring, analysing, integrating results) together contribute to mutual trust.

From additional literature, we identified a range of potential hindering factors, including lack of ownership of knowledge users, insufficient budget/structural funding and knowledge transfer, hobbyhorses of researchers, poor timing of results, unfavourable policy context (e.g. corona crisis, budget cuts, reorganisation), power relations and conflicts of interest. Facilitators found in the literature include the role of project leaders and/or policy officials, combination of different 'types' of knowledge, stakeholder commitment, embeddedness of the collaboration in the organisation/policy, and translation of knowledge (e.g. turning conclusions into practical recommendations and/or proposals).

Below, we describe the eight mechanisms for effective collaboration in more detail and focus on potential facilitating factors while also addressing some barriers. We illustrate the findings with examples from the different PKIs studied.

### Transdisciplinary collaboration

PKIs are an ideal setting for transdisciplinary collaboration; it involves research in which joint problem solving plays a key role [23], it deals with collaboration between scientific disciplines and domains, includes the active participation of societal stakeholders, and implies epistemic pluralism and science-internal reflexivity. An example is the academic collaborative network focusing on adolescents, which comprise a formal, long-term partnership between youth sector organisations, municipalities, universities, universities of applied science, parents and young people. These parties organise themselves regionally in a PKI that enables continuous interaction between them. The questions to be addressed are provided by adolescents and their parents, policymakers and health professionals. Researchers translate these questions into a (research) project. Together, the participants develop knowledge that can be directly used by health professionals, youth organizations and municipalities. To enhance the use of the knowledge developed within the academic collaborative network five learning networks were established in 2018. In a learning network, universities of applied science and practice organisations, work together, also with others like policy makers, experts, adolescents, lecturers, and researchers with the aim of sustainable exchange between training and practice organizations to enhance the quality of the work in the youth sector. Even though transdisciplinary collaboration is an important mechanism for collaboration [24], we found that the participation of for-profit organizations, citizens as well as collaboration between different domains (e.g. cure and care) are still at the early stages in the PKI studied. For example, citizens were involved in six out of the twenty PKIs: three academic collaborative networks (Public Health, Youth and Elderly Care), one disease/care-related network (Palliative Care Netherlands) and the living labs Sport and Exercise. Companies and health insurers are involved in two academic collaborative networks (Public Health, Integral approach to obesity), one consortium (Microplastics & Health) and the living labs Sport and Exercise.

### Shared ambition

Collaboration is effective when it provides something to all stakeholders involved [25]. It is essential that there is a shared ambition among the stakeholders operating in the PKI (bottom-up initiative). A shared ambition consists of a combination of strategies, goals and missions that are supported, pursued and adhered to within the PKI. To add societal value, stakeholders from different domains should be involved as early as possible in the process. The joint drafting of an impact pathway creates a shared picture of the (end) situation prompted by the interests of involved stakeholders. However, from our analysis it became clear that among the PKIs studied there is much emphasis on (international) scientific output and knowledge sharing, and less on creating a shared set of values (culture) to perpetuate knowledge implementation. One positive example is the collaborative network of organizations centred around pregnancy and birth. Before applying for funding from ZonMw, the relevant professional groups met at regional level to determine what goals they would like to achieve and what is needed to achieve the goals. According to those involved, this alone provided a big boost to better care for pregnant women and their babies. With the help of ZonMw grants, nine regional consortia were established, resulting in a nationwide pregnancy and birth network, collaborating with youth health services. The task of the Perinatal Care Board of the network is to stimulate and, where necessary, facilitate and organise knowledge exchange to support regional collaboration and to disseminate and make knowledge available to all stakeholders involved [26].

### Doing justice to everyone’s interests

Different interests may simultaneously play a role in a PKI: these could be organisational, individual and/or public interests. The interests determine how the knowledge issue at hand is being viewed, defined and perceived. Effective collaboration does as much as possible justice to everyone's interests. An example concerns the living labs Sport and Physical Activity, comprising fifteen municipalities and universities of applied science. With the help of a network grant, the partners and citizens mapped out their joint ambition. By involving citizens in the initial phase, the labs focus on citizens’ needs. Important insights emerge by analysing local issues together with citizens and giving them responsibility to finding solutions. Involving citizens in all steps of the process and adapting to their pace remains a challenge, especially when it comes to involving vulnerable groups. The labs use various tools for collaboration and engaging citizens, such as the flat puzzle (interaction by working on a puzzle together) [27] and kitchen table talks [28].

Effective collaborations aim for a win–win situation and include a continuous assessment of whether the collaboration creates value for involved stakeholders (individual, organisational and public). If there is room for mutual understanding of the interests of all stakeholders, the process will be smoother and more effective. To enable this open dialogue, fully participating (active) stakeholders, and transparent information exchange are essential. This should be defined from the end users perspective, to avoid mutually incorrect perceptions. Our analysis showed that organisational and individual interests have most often been taken into account in the PKI studied. However, there is insufficient commitment to public interests and insight into societal added value of each PKI.

### Personal relationships

Relationships between different stakeholders can affect effective collaboration. Possible opportunistic behaviour of stakeholders hinders knowledge collaboration. For example, researchers need to publish in journals that have a high-impact factors, and policymakers would prefer to receive concrete results on the short term. Our analysis shows that a weak project leader is a barrier for good collaboration. The role of the project leader is therefore essential for the relationship between/with parties and for the creation of a trusted environment. An example is an action programme of local initiatives, needed to provide an integrative approach, regarding troubled people in the province of Limburg. In this action programme learning networks are set up or expanded. Given the involvement of a large number of relevant parties including the police, safe houses, health insurers, and care providers, a strong project leader is necessary. The project leader fulfils a connecting role, manages cohesion and drives initiatives for a comprehensive approach targeted to troubled people. Thanks to the project leader partners are better able to reach out to each other, have a better understanding of what they can do for each other and have a better understanding of each other’s position. This lays the foundation for participatory partnerships in the future.

Our analysis also shows that there is insufficient insight regarding the challenges to collaboration, the existing knowledge base within PKI, and that less active partners may hinder collaboration. Connecting stakeholders enhances building personal relationships. The availability of a physical location is an important factor that contributes to a shared vision and personal contacts. Complementarity in thinking and doing is essential for effective collaboration. This requires attention to bringing together/synthesising from the outset necessary scientific knowledge from different disciplines (team science), experiential knowledge, professional knowledge (including practical experiences, research experiences outside the research setting and implementation skills) as well as artistic knowledge (creativity). An example in which art is used for connecting stakeholders and strengthening personal relations is the Beautiful Distress Madness meets Art initiative, part of Action Programme local initiatives for troubled people. The project takes a broad approach involving collaboration with people with mental health problems, their networks, artists, staff of mental health and community organisations, policymakers and adolescents and students. The recommendations have been discussed with all relevant parties, and collaboration has been used to further implement the results [29]. Finally, there are tools available such as the Involvement Matrix [30] that stimulate connecting with end users (e.g. patients, persons with disabilities, young people, parents, and relatives).

### Mutual trust

Trust is fundamental to effective collaboration and can be defined as “a psychological state of willingness to be vulnerable based upon positive expectations of the intentions or behaviour of another party in uncertain situations” [31]. Due to uncertainty, especially with regard to the future, trust is not a static element and can vary over the length of the relationship. It is apparent that mutual trust often stems from previous successful collaborations. Previous collaborations and bottom-up initiatives are enabling trust. Besides trust, an open attitude and mutual respect are important. For example, in the care network Palliative Care South-East collaboration between research partners, educational organizations, and health professionals is strengthened by joint agenda-setting and alignment in focus and priorities. Based on shared values and the development of a joint vision, projects and activities are carried out in the region, with trust forming the basis of collaboration [32].

### Professional organisation

The operational quality of the collaboration depends in part on the ability of the different stakeholders to engage organisationally and substantively, and the extent to which the collaboration can spur action [18]. Our analysis shows that there are different working routines/cultures (e.g. risk appetite) between research and practice (local government, healthcare professionals) that could potentially hinder this. A professional organisation is therefore essential for sustainable, effective collaborative partnerships. This involves establishing ground rules, procedures, agreements, relationships, ways of working (such as independent and agile working), (variable) roles and positions. We found that a supported culture, formalisation of responsibilities (e.g. declarations of intent and administrative consultations), (co)financing (in cash/in kind), effective (scientific) communication, a long-term vision, and thus long-term financing, are important facilitators. For example, experience from the regional oncology networks in the Netherlands shows that agreements on a national level are needed to explore—via large-scale experiments—how the networks financially can be sustained [33].

### Meaningful process

Facilitating and securing sustainable and learning infrastructures for development and implementation of knowledge are important factors for effective collaboration. This also applies to less protocolled and less complicated grant application processes as well as to strategy development of organisations. An example is the programme on how to effectively work in the youth sector, which is part of the academic collaborative network in the Youth sector. The aim of this programme is to increase, combine and disseminate knowledge on promoting the psychosocial development of children and adolescents. This is useful for the youth healthcare sector, local policy targeting prevention and/or clients at the interface of youth care/youth mental health/youth with mild intellectual disabilities. One of the projects within the programme focuses on professionals and their organisations (*n* = 21) to support them in developing a new way of working to transform the youth sector. Learning organisations is the key concept, requiring organisations to be flexible and innovative [34]. It is also important to ensure transparency of information and to harmonise the way in which knowledge infrastructures can be assessed. Data management (Open Science) facilitates communication and accountability with regard to research funding, analysis, and decision-making on resource allocation [35].

### Sufficient time and continuity

Our analysis shows that within PKIs there is a lack of processes that provide sufficient time, people and resources to learn to speak each other’s language, develop the soft skills needed for collaboration, have regular personal contact, room to experiment, reflect, implement and embed the collaboration in practice. The creation of connecting functions or linking pins (e.g., duo appointments, staff exchange) can be conducive factors to address these limitations. In addition, long-term programming based on knowledge questions and active steering of the funder promotes collaboration. An example of a PKI where linking pins have been instrumental is the academic collaborative network for Elderly Care South Limburg (AWO-ZL). This is a structural collaboration between a university, several healthcare organisations, a university of applied science and a secondary vocational education institution since 1998. Through scientific research, the AWO-ZL contributes to improving quality of life, quality of care and quality of work in the field of elderly care. To expand the AWO-ZL's impact, its infrastructure is being strengthened, based on a reciprocal ‘linking-pin’ construction: senior scientific staff and care professionals work in duo jobs at the organisations involved. Researchers and care professionals work together in an interdisciplinary way to generate and implement knowledge and they execute scientific research agendas set by the target population (elderly) and health professionals [36].

## Discussion

To our knowledge, this study is the first of its kind to map effective collaboration specifically in PKIs. As such, this research-on-research study was explanatory in nature. We studied what constitutes effective collaboration in long-term (research) partnerships, to what extent the mechanisms already appear in PKIs, and what this implies for the research governance of funders. Although our list of effective mechanisms for collaboration in PKIs may not be exhaustive, it is a first step to list these mechanisms, including potential facilitators and barriers so that they can be replicated and reported consistently.

Through a literature review we identified 8 mechanisms: transdisciplinarity; defining a shared ambition; doing justice to everyone’s interests; investing in personal relationships; a professional organisation or structure; a meaningful collaborative process; mutual trust, and sufficient time for and continuity of collaboration. In addition, we found that these mechanisms can either be enhanced or hindered by a combination of factors that relate to individuals involved in the partnership, their interrelationships and/or the working environment.

Based on the initial analysis of 20 selected PKIs we were able to identify all identified mechanisms in the literature. We also noted that there seems more emphasis on (international) scientific output and knowledge sharing, and less on creating a shared set of values (culture) and ambition to perpetuate knowledge implementation. Although it is generally recognised that citizen engagement and collaboration between different domains (e.g. care, welfare) is beneficial, this seems to be at its early start in PKIs studied. Furthermore, despite the intention of collaboration on equal grounds, critical reflection on the extent to which this actually happens was often not observed within the PKIs. Finally, a lack of understanding of the societal added value of PKIs in terms of knowledge utilisation and patient-related outcomes was indicated. The findings from the literature review and desk research were validated through several means, including an online survey of implementation experts, a Group Decision Room with lecturers, and interviews with key experts from different disciplines.

Taking these findings into consideration, organisers and funders of PKI’s, should more clearly stimulate joint development of knowledge, as well as proper knowledge utilisation. This requires cultural and behavioural change and social value orientation of all stakeholders within the knowledge ecosystem. To this end, we formulated a set of recommendations for funders, especially for ZonMw and their partners within the relevant knowledge ecosystem, using Nosek’s model [19] for structuring them. The main recommendations include:

**Table:** 

Policy: Make it required
• (Continue to) stimulate Open Science and the national Recognition and Rewards programme [37] • Focus on 'fund and fellowship' instead of ‘fund and forget’ • Allocate R&D budget for reflection on and improvement of funding practices (research-on-research) • Develop a systematic approach to monitor, report, reflect and evaluate practices, experiences and achieved impact (desired/unwanted, expected/unexpected) from different perspectives
Incentives: Make it rewarding
• Develop standardized assessment processes to identify and prioritize transdisciplinary collaborations (e.g., interviews in the proposal stage about leadership and teamwork) • Set clear funding conditions to enhance effective knowledge collaboration (e.g., need for continuous interaction/participation during the research process with/from research funders) • Commit to long-term programming and funding (beyond five years) based on analysis of knowledge questions and knowledge infrastructure(s) in the respective field(s) • Organize separate funding calls for infrastructures targeting societal impact, with a focus on learning systems • Organize site visits, kick-offs and national peer/project leader meetings with ‘best persons’ • Undertake and support ongoing, collaborative translation of research recommendations into policy, practice and education
Communities: Make it normative
• Provide training to referees and committee members on cocreation • Provide training to project leaders on effective communication and change management
User interface: Make it easy
• Stimulate hybrid research (e.g., combining systematic reviews and primary research) in which different types of knowledge and expertise from different disciplines, domains and fields are integrated • Deploy implementation professionals and knowledge coaches to facilitate knowledge sharing and translation of results into policy, practice and education • Allocate sufficient budget for dissemination (e.g. knowledge platforms) and implementation of results that are targeting end users (e.g. via learning networks)
Infrastructure: Make it possible
• Ensure proper reporting and provision of information on websites and other dissemination channels • Be transparent about the quality of (steering) information, sources and how to manage (competing) interests • Perform knowledge syntheses and focus on systematic knowledge building (content and collaboration)

These recommendations are meant as a roadmap towards the realisation and demonstration of societal impact of health research in the upcoming years.

## Conclusions

This exploratory research-on-research study shows that there are eight different mechanisms (‘how and why’) that are—in conjunction with each other-decisive for effective collaboration in PKIs: transdisciplinary collaboration; defining a shared ambition; doing justice to everyone's interests; investing in personal relationships; a professional organisation or structure; a meaningful collaborative process; mutual trust, sufficient time for and continuity of collaboration. Several factors (‘what’) may hinder (e.g., lack of ownership or structural funding) or facilitate (e.g., stakeholder commitment, embeddedness in an organisation or policy) effective collaboration in research partnerships. As such no-one-size-fits all impact pathway was identified for the PKIs studied. It is clear that the challenge for organisers and funders of PKIs is to properly embed joint development and utilisation of knowledge in policy, practice, education and/or (further) research. For this reason, we have formulated several recommendations that could (jointly) be taken forward by the respective organizations. For funders like ZonMw, it does not just mean more commitment to long-term programming and funding. It involves a completely different way of working. To support transition and transformation funders must actively engage in the partnerships they fund, reflect together on what goes well and what does not along the road, and continuous learn from this for the next steps to be taken. It is encouraging to know that ZonMw has recently started to internally discuss these recommendations to enhance its public performance.

### Supplementary Information

Additional file 1.

## Data Availability

The source of data and materials are mentioned in the manuscript, in support of the findings. The materials and data supporting the findings of the article is available on Surfdrive, and are available from the corresponding author, [WR], on special request.

## Abbreviations

ACN: Academic collaborative centres
AESIS: Advancing and Evaluating the Societal Impact of Science
AWO-ZL: Network for Elderly Care South Limburg
C: Consortium
CN: Disease-related/care networks
EIE: European Implementation Event
EViR: Ensuring Value in Research
GDR: Group Decision Room
KP: Knowledge portal
LL: Living labs
LN: Learning networks
NIC: Nederlands Implementatie Collectief
PKI: Participatory knowledge infrastructure
ZonMw: Netherlands Organization for Health Research and Development

## Footnotes

1 https://gallery.mailchimp.com/7fa42547078f2cac7d96896f5/files/54710d19-6a40-4f27-a8c9-c3a15a010a59/Wendy_paper.pdf.
