# Supplementary material for: How to strengthen societal impact of research and innovation? Lessons learned from an explanatory research-on-research study on participatory knowledge infrastructures funded by the Netherlands Organization for Health Research and Development
Source: Health Res Policy Syst. 2024 Jul 8;22:81. doi: 10.1186/s12961-024-01175-x (PMC11229179; doi:10.1186/s12961-024-01175-x)
Supplement: Supplementary file 1 — Additional file 1. [file 12961_2024_1175_MOESM1_ESM.docx]

Additional file 1 Analysis tool

**I Type of participative knowledge infrastructure (PKI)**

- **Content/purpose:** What is the focus of the PKI? Has an impact pathway^^[[1]](#footnote-1)^^ been drafted?
- **Relations:** Who are involved in the PKI?^^[[2]](#footnote-2)^^
- **Structure / process:**
- To what extent is the collaboration effective with regard to the knowledge transfer and utilization of knowledge?
- Which routes to knowledge utilization have been followed? (defining impact pathway(s))

**II Which barriers to collaboration can be distinguished?**

*Barriers could relate to individuals involved in the partnership (individual factors), their interrelationships (relational factors) and/or the working environment (contextual factors). Examples are a lack of ownership of knowledge users; insufficient or no structural funding for knowledge transfer; 'hobbyhorses' of researchers; poor timing of disseminating results; unfavourable policy context (e.g. corona crisis)*

***[Pay specific attention to productive interactions***^^[[3]](#footnote-3)^^***]***

**III Which facilitators to collaboration can be distinguished?**

*Facilitators could relate to individuals involved in the partnership (individual factors), their interrelationships (relational factors) and/or the working environment (contextual factors). Examples include the role of project leaders and/or civil servants; shared interests; combination of different 'types' of knowledge; stakeholder commitment; embedding of collaborative structure in the organisation or policy; translation of knowledge).^^[[4]](#footnote-4)^^*

***[Pay specific attention to productive interactions***^^[[5]](#footnote-5)^^***]***

**IV Role of ZonMw as funder:** How can ZonMw facilitate/stimulate effective collaboration? This can relate to different aspects, see figure below.


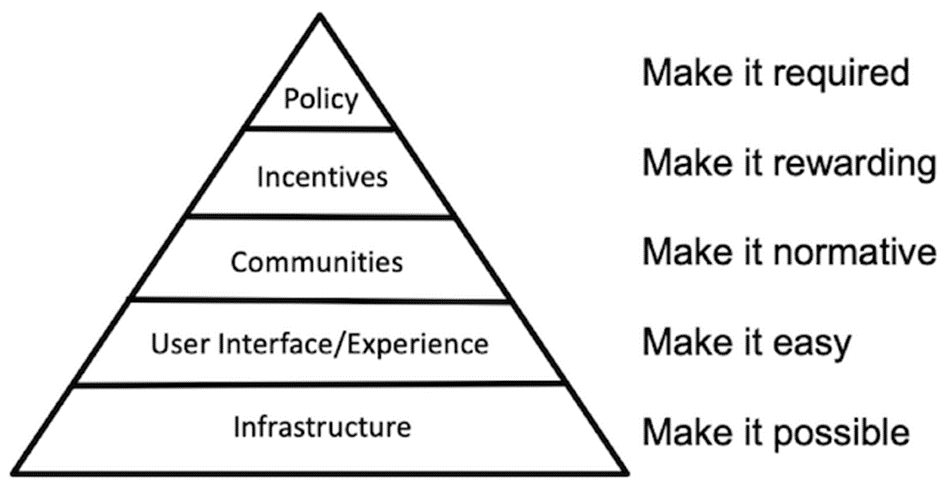


Source: Nosek, 2019^[[6]](#footnote-6)^

**V Other remarks**

1. A description of what you wish to achieve with your project and why, when, how and with whom you wish to achieve it [↑](#footnote-ref-1)
2. Categories/fields: practice (prevention/cure/care/welfare), policy (local/regional/national/international), research/science, education, patients/citizens, business, other [↑](#footnote-ref-2)
3. Productive interactions are factors of which we know that they increase the chances of knowledge utilisation in practice, policy, education and/or further research. These are collaboration with relevant stakeholders; co-financing; delivery of usable knowledge products and targeted dissemination and implementation activities [↑](#footnote-ref-3)
4. Hoekstra F, Mrklas KJ, Khan M. et al. A review of reviews on principles, strategies, outcomes and impacts of research partnerships approaches: a first step in synthesising the research partnership literature. Health Res Policy Sys. 2020; doi.org/10.1186/s12961-020-0544-9.; Zych MM, Berta WB, Gagliardi AR. Conceptualising the initiation of researcher and research user partnerships: a meta-narrative review. Health Res Policy Syst. 2020; doi: 10.1186/s12961-020-0536-9. [↑](#footnote-ref-4)
5. Productive interactions are factors of which we know that they increase the chances of knowledge utilisation in practice, policy, education and/or further research. These are collaboration with relevant stakeholders; co-financing; delivery of usable knowledge products and targeted dissemination and implementation activities [↑](#footnote-ref-5)
6. Nosek B. Changing a research culture. In: Strategy for culture change. Blog, 11 juni 2019. https://www.cos.io/blog/strategy-for-culture-change. Accessed 6 April 2023. [↑](#footnote-ref-6)
